# N-Acetylcysteine normalizes brain oxidative stress and neuroinflammation observed after protracted ethanol abstinence: a preclinical study in long-term ethanol-experienced male rats

**DOI:** 10.1007/s00213-023-06311-z

**Published:** 2023-01-28

**Authors:** Sandra Fernández-Rodríguez, María José Cano-Cebrián, Claudia Esposito-Zapero, Salvador Pérez, Consuelo Guerri, Teodoro Zornoza, Ana Polache

**Affiliations:** 1 Department of Pharmacy and Pharmaceutical Technology and Parasitology, Faculty of Pharmacy, University of Valencia, Burjassot, 46100 Valencia, Spain; 2 Department of Physiology, Faculty of Pharmacy, University of Valencia, Burjassot, 46100 Valencia, Spain; 3 Department of Molecular and Cellular Pathology of Alcohol, Príncipe Felipe Research Center, Valencia, Spain

**Keywords:** N-acetylcysteine, Protracted abstinence, Glutamate transporters, Oxidative stress, Neuroinflammation, Ethanol relapse

## Abstract

**Rationale:**

Using a preclinical model based on the Alcohol Deprivation Effect (ADE), we have reported that N-Acetylcysteine (NAC) can prevent the relapse-like drinking behaviour in long-term ethanol-experienced male rats.

**Objectives:**

To investigate if chronic ethanol intake and protracted abstinence affect several glutamate transporters and whether NAC, administered during the withdrawal period, could restore the ethanol-induced brain potential dysfunctions. Furthermore, the antioxidant and anti-inflammatory effects of NAC during abstinence in rats under the ADE paradigm were also explored.

**Methods:**

The expression of GLT1, GLAST and xCT in nucleus accumbens (Nacc) and dorsal striatum (DS) of male Wistar was analysed after water and chronic ethanol intake. We used the model based on the ADE within another cohort of male Wistar rats. During the fourth abstinence period, rats were treated for 9 days with vehicle or NAC (60, 100 mg/kg; s.c.). The effects of NAC treatment on (i) glutamate transporters expression in the Nacc and DS, (ii) the oxidative status in the hippocampus (Hip) and amygdala (AMG) and (iii) some neuroinflammatory markers in prefrontal cortex (PFC) were tested.

**Results:**

NAC chronic administration during protracted abstinence restored oxidative stress markers (GSSG and GGSH/GSH) in the Hip. Furthermore, NAC was able to normalize some neuroinflammation markers in PFC without normalizing the observed downregulation of GLT1 and GLAST in Nacc.

**Conclusions:**

NAC restores brain oxidative stress and neuroinflammation that we previously observed after protracted ethanol abstinence in long-term ethanol-experienced male rats. This NAC effect could be a plausible mechanism for its anti-relapse effect. Also, brain oxidative stress and neuroinflammation could represent and identify plausible targets for searching new anti-relapse pharmacotherapies.

## Introduction

According to the World Health Organisation “Global Status Report on Alcohol and Health” 5,1% of the adult population worldwide suffered from alcohol use disorder (AUD) and around 3 million persons have died in 2016 due to ethanol misuse (World Health Organization, 2018). Relapse is one of the most severe handicaps of this psychopathological condition since it has been reported that patients can relapse even after several months or years of abstinence (Weiss et al. 2001; Barrick & Connors, 2002). Unfortunately, nowadays there are no completely successful pharmacotherapies to prevent ethanol relapse (Spanagel & Vengeliene, 2013; Reilly et al. 2014; Cannella et al. 2019). In fact, relapse rates remain high even for patients receiving currently available treatments (National Institute on Alcohol Abuse and Alcoholism, 2021). Although in the last decade a wealth of studies has investigated the neurobiological mechanisms involved in the various phases of AUD, there are still some gaps lingering in the literature (Gipson et al. 2021). Consequently, the literature underlines the need of unravelling molecular mechanisms linked to the relapse behavior displayed by vulnerable individuals. These findings would have the potential to improve AUD treatments (Cannella et al. 2019; Zhang et al. 2019). In this context, by using the alcohol deprivation effect (ADE) model in male rats, our group recently reported biochemical differences between rats that repeatedly displayed relapse-like drinking behavior and those who did not. Concretely, augmented oxidative stress levels and increased expression of immunoinflammatory markers such as IL-1β and TNFα were found after a protracted abstinence period (3-week) only in rats that repeatedly manifested relapse behavior, possibly being a key in the induction of the craving that will lead to the relapse process (Fernández-Rodríguez et al. 2022). In accordance with our results, a recent review stated that the development of anti-relapse pharmacotherapies should target neuroinflammation and oxidative stress alterations underlying AUD (Namba et al. 2021).

N-Acetylcysteine (NAC), a pleiotropic drug associated with antioxidant, glutamatergic and anti-inflammatory capabilities (Oka et al. 2000; Kupchik et al. 2012; Badisa et al. 2013), has shown promising results in preclinical studies as treatment for several substance used disorders (SUDs) in the case of ethanol (Quintanilla et al. 2016; Lebourgeois et al. 2019), cocaine (Ducret et al. 2016; Woodcock et al. 2021), heroin (Hodebourg et al. 2019) and nicotine (Quintanilla et al. 2018)). In fact, a recent study conducted in our laboratory has also demonstrated the ability of NAC to prevent ethanol relapse using the ADE model, a high face, predictive and ecological validity model in the preclinical setting (Cano-Cebrián et al. 2021). Although some studies have analysed the effects of NAC on brain glutamate neurotransmission, oxidative status or inflammatory markers, the results are not always coincident and the understanding of the molecular mechanisms underlying NAC effects in SUD remain incomplete (Smaga et al. 2021). In this paper, we wanted to investigate brain glutamate neurotransmission, oxidative status or inflammatory markers in a period where NAC treatment is efficient to decrease relapse.

A paradoxical result of our previous studies indicated that in rats that manifested relapse-like drinking behaviour, ethanol re-introduction was able to normalize the altered oxidative status and neuroinflammation markers observed after a protracted (21 days) ethanol abstinence (Fernández-Rodríguez et al. 2022). Consequently, we further assessed the underlying mechanism of action of NAC in ethanol relapse, analysing its neurobiological effects during the abstinence period, in the absence of ethanol that potentially interferes in the altered neuroimmune and oxidative status and would mask NAC effects.

According to the literature, another potential mechanism that may underlie the observed anti-relapse effect of NAC could be related to the alteration of glutamate homeostasis, which is also relevant in drug relapse (Kalivas 2009; Alasmari et al. 2018). In fact, it has been shown that astrocytic dysfunction in regulating both the basal glutamate levels as well as clearance of synaptic glutamate is a primary mechanism whereby cues associated with drugs of abuse can drive relapse (Kalivas 2009; Griffin et al. 2015; Scofield et al. 2016). Moreover, the effects of chronic alcohol consumption and the effect of abstinence on astroglial high affinity glutamate transporters and/or the glutamatergic signaling machinery is not completely understood so far. It should be considered that abstinence represents a critical period of vulnerability within the addiction cycle, and the altered glutamatergic homeostasis sequelae of protracted ethanol abstinence remains poorly known (Namba et al. 2021). To date, very few studies have examined how glutamate transporters are altered across a protracted period of abstinence compared to chronic ethanol exposure, and if such changes are causally linked to relapse-like drinking behaviour (Ding et al. 2013). Some studies suggest that pharmacological agents acting upstream of glutamate transport systems (e.g., GLT-1 and xCT) to influence broader aspects of glial function also disrupt drug reinstatement and relapse (Kalivas 2009; Sari et al. 2013), giving a plausible explanation for the reported anti-relapse NAC effects. Nonetheless, at present the effect of NAC in these transporters during a protracted abstinence is not well characterized.

Therefore, the aim of this study was to explore the potential mechanisms by which NAC could probably exert its effects during long-term abstinence (i.e.,, when ethanol is not present, avoiding ethanol interferences) to prevent the ethanol relapse-like drinking behaviour in the ADE preclinical model. To do so, we treated a cohort of rats under this paradigm with vehicle, NAC 60 mg/kg or NAC 100 mg/kg during the deprivation period and examined different brain areas that are highly affected in AUD (Chefer et al. 2011; Elibol-Can et al. 2011; Roberto et al. 2004): neuroinflammation in prefrontal cortex (PFC) and oxidative stress imbalance in the hippocampus (Hip) and amygdala (AMG). Likewise, a further goal of the present research was to examine the effect of protracted ethanol withdrawal on two synaptic glutamate transporters (GLT-1 and GLAST) and cysteine/glutamate antiporter in two areas of the striatum, concretely nucleus accumbens (Nacc) and dorsal striatum (DS). We will further evaluate whether NAC could act restoring the potential alterations. Understanding the mechanistic properties of NAC to prevent alcohol relapse after a protracted period of abstinence could help to optimise the present pharmacotherapies and to identify future effective treatments.

## Experimental procedures

### Animals

Thirty male Wistar rats purchased from ENVIGO (Barcelona, Spain) were used. These animals were previously used for behavioural and some biochemical studies (Fernández-Rodríguez et al. 2022) as explained below. All animals, weighing 356 ± 27 g at the beginning of the experiment, were housed in individual cages in a temperature- and humidity-controlled room with a 12-h inverted light/dark cycle (on 22:00, off 10:00). All the procedures were performed in accordance with European Council Directive 2010/63/EU for animal experiments, Spanish laws (RD 53/2013) and animal protection policies. The Animal Care Committee of the University of Valencia and the Regional Government approved and authorized all experiments.

### Drugs

Ethanol drinking solutions at different concentrations (5, 10 and 20%) were prepared from 96% ethanol v/v (Scharlau S.A., Spain). NAC was purchased from Sigma-Aldrich Quimica, S.A. (Spain) and was freshly dissolved before use at 100 mg/ml in phosphate buffer 0.4 M (pH was adjusted to 7.2 with NaOH).

### Long-term voluntary alcohol drinking procedure

**Fig. 1:**
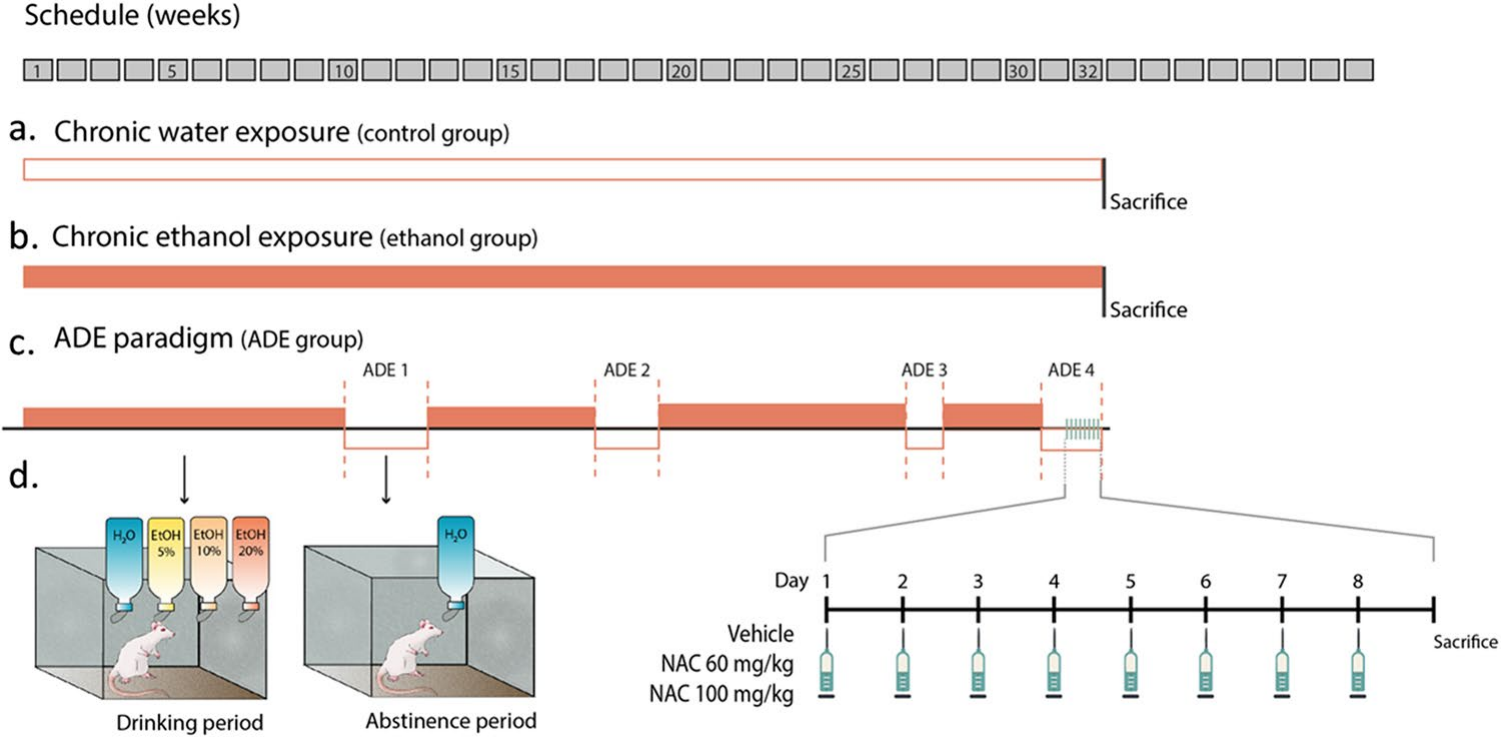
Timeline paradigm depicting the different experimental groups. On top, weeks are represented by grey squares while in a, b and c water and ethanol consumption periods are represented in white and orange, respectively. (**a**) Animals were chronically exposed to water consumption (*n* = 9) (Control group). (**b**) Animals were chronically exposed to ethanol consumption (*n* = 6) under the four-bottles free choice paradigm (Ethanol group). (**c**) Animals under the ADE paradigm (*n* = 15). During the fourth abstinence period these animals were subcutaneously injected for nine consecutive days, once a day, with vehicle (Vehicle group), NAC 60 mg/kg (NAC 60 mg/kg group) or NAC 100 mg/kg (NAC 100 mg/kg group). After this period, all animals were immediately sacrificed to obtain their brains

The experimental design and timeline are illustrated in Fig. 1. All animals were individually housed and had free access to standard food. As pointed out in our previous paper (Fernández-Rodríguez et al. 2022), a cohort of 15 rats was randomly assigned to two separate groups: (A) Control group: animals subjected to a long-term voluntary ethanol drinking procedure, i.e., they had continuous access to tap water (*n* = 9) and (B) Ethanol group: animals that had free access to 4 drinking bottles (tap water and 5%, 10% and 20% (v/v) ethanol dilution) (*n* = 6). After 32 weeks, all rats were euthanized to remove their brains. An additional cohort of 15 rats was subjected to a long-term voluntary ethanol drinking procedure but with repeated deprivation phases (ADE model). N.B. ADE is a transient increase in the alcohol intake over basal values following a period of deprivation which correlates with the loss of control associated with the alcohol relapse-like drinking behaviour (Spanagel 2017). During this procedure, animals had voluntary continuous access to tap water and 5%, 10% and 20% (v/v) ethanol solutions and were subjected to four random deprivation periods (total duration 32 weeks). This paradigm had previously been used and validated by our research group (Orrico et al. 2013, 2014; Cano-Cebrián et al. 2021; Fernández-Rodríguez et al. 2022). Specifically, rats were subjected to four drinking (6 ± 2 weeks) and four deprivation (2 ± 1 weeks) periods. The duration of each period was deliberately irregular to avoid behavioural adaptations (Vengeliene et al. 2005) (Fig. 1c). Every time bottles were weighed to determine the intake, their position was changed to avoid location preferences. The 15 rats came from a wider cohort of animals (*n* = 30) that were characterized in our previous research (Fernández-Rodríguez et al. 2022). Concretely, all these rats repeatedly displayed the relapse-like drinking behaviour. The pharmacological studies with NAC were initiated at the end of the fourth alcohol deprivation period as detailed in the following.

### NAC treatment

Pharmacological treatment was initiated on the 12^th^ day of the fourth abstinence period (Fig. 1c). Animals were randomly assigned to three experimental subgroups, which were subcutaneously injected with vehicle, NAC 60 mg/kg or NAC 100 mg/kg (*n* = 5 per group) once a day for 9 consecutive days. NAC doses and schedule were selected according to those used in our previous paper (Cano-Cebrián et al. 2021). Twenty-four hours after the last administration, rats were euthanized to remove their brains. Hence, all rats included in these three experimental subgroups were sacrificed after a protracted abstinence (3 weeks).

To sum up, five different experimental groups were designed and tested according to the different techniques described below: Control (*n* = 9), Ethanol (*n* = 6), Vehicle (*n* = 5), NAC 60 mg/kg (*n* = 5) and NAC 100 mg/kg (*n* = 5) (Fig. 1).

### Tissue collection

Brains were extracted and immediately frozen and stored at − 80 °C. Subsequently, brain regions of interest: Nacc, DS, Hip, AMG and PFC were dissected according to the rat brain stereotaxic atlas (Paxinos and Watson 2007). Afterwards, selected areas were separately stored to analyse the parameters of interest by Mass Spectrometry, Western Blot or Reverse Transcription PCR (RT-PCR). The present research focuses on brain areas that are highly affected by ethanol consumption and abstinence-induced damage (Chefer et al. 2011; Elibol-Can et al. 2011; Roberto et al. 2004; Zhang et al. 2019).

### Western blot: determination of GLT-1, GLAST and xCT expression

This technique was used to measure the expression levels of GLT-1, GLAST and xCT in Nacc and DS from animals belonging to all experimental groups. Samples were homogenized with RIPA lysis buffer (SDS 0,1%, Igepal CA-360 1%, sodium deoxycolate 0,5% and protease inhibitors cocktail 1% in PBS). Homogenates were kept on ice for 30 min and centrifuged at maximum r.p.m. for 15 min. The supernatant was collected, and the protein content was quantified with Bradford Reagent (Bio-Rad). Protein samples were separated by 10% SDS-PAGE gels and transferred to nitrocellulose membrane. Transference was performed with a semi-dry system (Bio-Rad Trans-Blot® TurboTM) for 25 min at 25 V. Then, membranes were blocked 1 h at room temperature with blocking solution (5% non-fat milk in TBS containing 0.1% of Tween-20 (TBS-T)). Primary rabbit antibodies were diluted in blocking solution and incubated overnight at 4 °C: anti-GAPDH (1:2000 #G9545 Sigma-Aldrich), anti-GLT-1 (1:5000 ab41621 from Abcam), anti-xCT (1:1000 ab175186 from Abcam), and anti-GLAST (1:2000 NB100-1869 from NovusBio). Membranes were washed with TBS-T and incubated with HRP-conjugated secondary antibody diluted in blocking solution (Rabbit 1:1000–1:3000 from Bio-Rad) for 1 h at room temperature. TBS-T and TBS were used to wash membranes before band detection. Chemiluminescence was visualized using Clarity Max substrate (Bio-Rad). The intensity of the obtained bands was quantified with the α-Ease FC software, version 4.0.0 (Alpha Innotech Corporation). Protein levels were normalized with GAPDH signal which was used as a loading control. Obtained values in the different groups were expressed as percentage of that obtained in the control group.

### Determination of GSH and GSSG levels by mass spectrometry

In our previous study, we analysed the oxidative status in control and ethanol rats in the Hip and AMG (Fernández-Rodríguez et al. 2022). In the present study, we focus on the effect of NAC during the abstinence period on the GSH and GSSG levels in the same areas (vehicle, NAC 60 mg/kg and NAC 100 mg/kg groups). After the dissection of Hip and AMG, tissues were homogenized in phosphate buffered saline (PBS) and 10 mmol/L N-ethylmaleimide (NEM) (Sigma-Aldrich, St. Louis, MO, USA) (pH 7.0), with a tissue-buffer ratio of 1:4. Then, perchloric acid solution was added to obtain a final concentration of 4% and samples were centrifuged at 11,000 rpm for 15 min at 4 °C. Supernatants were injected into the chromatographic system (UPLC-MS/MS).

The chromatographic system consisted of a Micromass QuatroTM triple-quadrupole mass spectrometer (Micromass, Manchester, UK) equipped with a Zspray electrospray ionization source operating in the positive ion mode with a LC-10A Shimadzu (Shimadzu, Kyoto, Japan) coupled to the MassLynx 4.1 software for data acquisition and processing. Samples were analysed by reversed-phase UPLC as previously reported (Fernández-Rodríguez et al. 2022). Calibration curves were obtained using twelve-point (0.01–100 mmol/l) standards (purchased from Sigma-Aldrich, St. Louis, USA) for each compound. The concentrations of metabolites were expressed as nmol/mg of protein.

### Reverse transcription PCR: determination of neuroinflammatory mediators

The gene expression levels of different inflammatory mediators, such as TNF-α, IL-6, IL-1β, iNOS, Nfκβ, HMGB1 and NLRP3, were determined in the PFC of the rats assigned to the following groups: vehicle, NAC 60 mg/kg and NAC 100 mg/kg, since the determination in control and ethanol group had already been performed and reported. RNA extraction, cDNA synthesis and RT-PCR conditions used had previously been described and reported by our group (Fernández-Rodríguez et al. 2022). The relative expression ratio of a target/reference gene was calculated according to the Pfaffl equation (Pfaffl 2001). Housekeeping cyclophilin A (PPIA) was used as an internal control. The sequences of primers used in this study are gathered in Table 1.

**Table 1:** Primer sequences used

Gene	Forward primer (5′-3′)	Reverse primer (5′-3′)
*PPIA*	TGTGCCAGGGTGGTGACTTT	CGTTTGTGTTTGGTCCAGCAT
*IL1Β*	CAGCAGCATCTCGACAAGAG	CATCATCCCACGAGTCACAG
*IL6*	TGTGCAATGGCAATTCTGAT	CGGAACTCCAGAAGACCAGAG
*TNFΑ*	GGTGGGCTGGGTAACAAGTA	AGGGACAAACCACAATATAGGAAAA
*HMGB1*	ATCTAAATACGGATTGCTCAGGAA	AGGGACAAACCACAATATAGGAAAA
*NFΚΒ*	CAAGAGTGACGACAGGGAGAT	GCCAGCAGCATCTTCACAT
*NLRP3*	CCCTCATGTTGCCTGTTCTT	TCCAGTTCAGTGAGGCTCTG

Fluorescence was recorded in the annealing/elongation step in each cycle. To check the specificity of the primers, a melting curve analysis was performed at the end of each PCR. All these procedures had previously been validated in our laboratory (Vallés et al. 2004; Alfonso-Loeches et al. 2014; Ureña-Peralta et al. 2020).

### Statistical analysis

A power analysis was performed that revealed that a sample size of *N* = 4–5/group was determined necessary to detect differences in the key variables at an *α* level of *p* < 0.05 and 80% power. All data were tested for normality and homoscedasticity. Then, experimental data, i.e. levels of GSH, GSSG, GSH/GSSG, and different protein and mRNA expression were analysed using two different analysis of variance (one-way ANOVA). On the one hand, the influence of ethanol consumption or ethanol abstinence was studied through the comparison among data derived from Control, Ethanol, and Vehicle group. On the other hand, the comparison among data from Vehicle, NAC 60 mg/kg and NAC 100 mg/kg allowed to explore the effect of NAC under abstinence conditions. Post hoc comparisons were performed through the application of Tukey’s test when appropriate. The significance level was always set at *p* = 0.05. All data are presented as mean ± standard error (SE). All the analysis were carried out using GraphPad Prism, version 8.0.1 (GraphPad Software Inc).

## Results

The rats used in the following experiments repeatedly displayed the ADE phenomenon. Concretely, the mean basal ethanol intake before the abstinence period was 1.94 ± 0.15 g/kg/day and rose to 2.90 ± 0.24 g/kg/day after ethanol reintroduction.

### Glutamate homeostasis

**Fig. 2:**
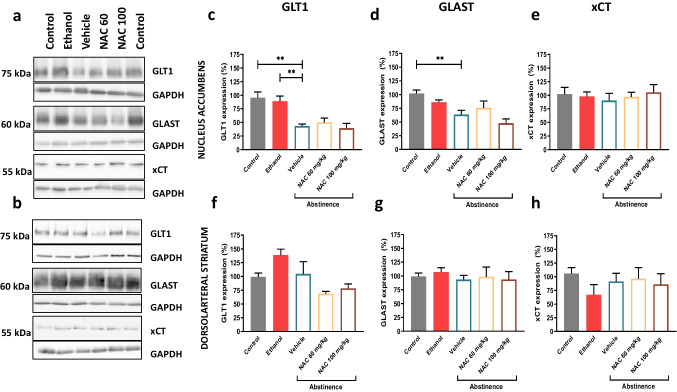
Glutamate transporters expression after (i) chronic ethanol intake, (ii) prolonged abstinence and (iii) effect of NAC treatment during prolonged abstinence. Representative Western blot images obtained in Nacc (**a**) and DS (**b**). Protein levels of GLT1 (**c**), GLAST (**d**) and xCT (**e**) in Nacc, and protein levels of GLT1 (**f**) GLAST (**g**) and xCT (**h**) in DS. Data are represented as % of control group value and expressed as mean ± SE. Each graph represents protein levels from rats under different conditions: animals exposed to water consumption (control) in grey; animals chronically exposed to ethanol consumption (ethanol) in red; and animals under abstinence conditions treated with vehicle (blue framed bar), NAC 60 mg/kg (orange framed bar) or NAC 100 mg/kg (brown framed bar). For data analysis, two different one-Way ANOVA followed by Tukey’s post hoc were applied. Asterisk (**p < 0.01]) indicates significant differences among “control”, “ethanol” or “vehicle” groups. No statistical differences were detected among “vehicle”, “NAC 60 mg/kg” and “NAC 100 mg/kg”

To assess the potential alteration in glutamate homeostasis during chronic ethanol exposure as well as during protracted abstinence, the expression of the main proteins involved in brain glutamate homeostasis, i.e., GLT-1, GLAST and xCT, was measured in Nacc and DS. In the case of GLT-1 in Nacc (Fig. 2c), one-way ANOVA detected statistical differences among control, ethanol and vehicle groups (F = 2, 14 = 9.433; *p* = 0.0025). The post hoc analysis revealed that GLT-1 expression was not altered after chronic ethanol consumption when compared with respect to the control group (*p* = 0.8544). However, interestingly, GLT-1 levels were significantly decreased (around 45%) during the abstinence period (vehicle group) when compared not only with respect to the control (*p* = 0.0032) but also with the ethanol group (*p* = 0.0087). The downregulation detected during the withdrawal period was neither reverted by NAC 60 mg/kg nor by 100 mg/kg treatment, as one-way ANOVA did not detect statistical differences (F(2,11) = 0.6076; *p* = 0.5619). Regarding GLAST levels in Nacc (Fig. 2d), one-way ANOVA also revealed significant differences (F(2,14) = 9.434; *p* = 0.0025). Thus, although when compared with control group, GLAST expression remained invariable after chronic ethanol intake (*p* = 0.6550), a significant reduction of around 37% was detected under abstinence conditions (*p* = 0.0018) and a nearly significant reduction when compared with the ethanol group (*p* = 0.055). As in the case of GLT-1, the administration of NAC was not able to counteract this effect during abstinence (F(2,11) = 1.848; *p* = 2033). Concerning xCT expression in Nacc, no statistical differences were detected after chronic ethanol intake or alcohol abstinence (F(2,13; = 0.2801; *p* = 0.7601) (Fig. 2e). In DS, neither GLT-1 (F(2,14) = 2.633; *p* = 0.1070) (Fig. 2f), nor GLAST (F(2,14) = 0.8511; *p* = 0.4479) (Fig. 2g) nor xCT expression (F(2,14) = 1.717; *p* = 0.2154) (Fig. 2h) were altered under any experimental conditions in the DS.

### Brain oxidative status

**Fig. 3:**
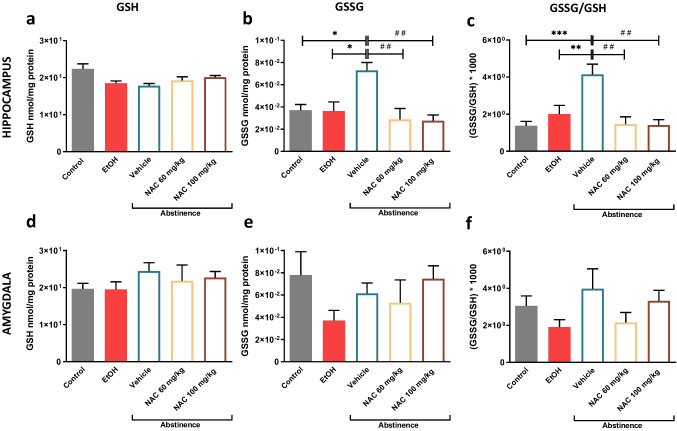
Oxidation markers after (i) chronic ethanol intake, (ii) prolonged abstinence and (iii) effect of NAC treatment during prolonged abstinence. GSSG, GSH levels and GSSH/GSH ratio (**a**–**c**) in Hip or amygdala (**d**–**f**). Data are expressed as nmol/mg of protein and as mean ± SE. Each graph represents metabolite levels from rats under the different experimental conditions described in Fig. 1. The colour legend is the same as the one detailed in Fig. 2. For data analysis, two different one-way ANOVA followed by Tukey’s post hoc were applied. Asterisks (* *p* < 0.05; ** *p* < 0.01; *** *p* < 0.001) indicates significant differences among “control”, “ethanol” or “vehicle” groups, and the hash symbol (^##^*p* < 0.01) indicates significant differences among “vehicle”, “NAC 60 mg/kg” or “NAC 100 mg/kg groups

We further evaluated the potential ability of NAC, administered during abstinence, to restore the brain redox imbalance previously detected along this period. For this aim, levels of oxidized (GSSG) and reduced (GSH) glutathione were measured in two brain regions: Hip and AMG (Fig. 3). Although data from control and ethanol groups had previously been reported (Fernández-Rodríguez et al. 2022), they have been plotted together with the present results as well as included in the statistical analysis for comparative purposes. Considering that the GSSG/GSH ratio is one of the most common indices of oxidative stress, it was also calculated. The most remarkable results were obtained within the Hip (Fig. 3c), where NAC was able to blunt the large rise in the GSSG/GSH ratio detected when animals are experiencing prolonged abstinence (F(2,9) = 13.500; *p* = 0.0020). Concretely, the GSSG/GSH ratio observed during abstinence rose to 300% and 207% in comparison with the control (*p* = 0.0006) or ethanol (*p* = 0.0099) groups, respectively. This increase in oxidative stress was normalized by both NAC 60 mg/kg (*p* = 0.0038) or NAC 100 mg/kg (*p* = 0.0026) administration during abstinence (Fig. 3c). It is important to note that, as can be observed in Fig. 3a, no significant changes in GSH levels were detected under any experimental condition (F(2,15) = 3.823; *p* = 0.0827). On the contrary, statistical analysis showed significant differences in GSSG values (F(2,15) = 5.768; *p* = 0.0139) (see Fig. 3b). From the general observation of Fig. 3b and c, it could be noted that GSSG levels display similar trends as those observed in the GSSG/GSH ratio. Accordingly, during abstinence, GSSG values significantly peaked when compared with the control (*p* = 0.0156) or ethanol (*p* = 0.02) group. Importantly, the administration of either of the NAC doses assayed in abstinent rats rapidly alleviated this rise and restored GSSG levels when compared to the vehicle group (*p* = 0.0091 and 0.0060, respectively).

Concerning data obtained in the AMG, one-way ANOVA did not detect statistical differences when GSH (F (2,13) = 1.900, *p* = 0.1888), GSSG (F (2,14) = 1.325, *p* = 0.2972) or GSSG/GSH ratio (F (2, 12) = 2.254, *p* = 0.1475) were analysed after chronic alcohol exposition or ethanol abstinence (Fig. 3d, e and f). Moreover, NAC administration did not modify neither GSH (F (2,10) = 0.2151, *p* = 0.8101) nor GSSG (F (2,10) = 0.5999, *p* = 0.5675) nor GSSG/GSH ratio (F (2, 10) = 2.254, *p* = 0.1475).

Overall, these findings suggest that under our experimental conditions, oxidative status in Hip is altered during abstinence and both NAC doses, chronically administered during this period, are able to alleviate this imbalance, leading it to basal conditions.

### Neuroinflammatory status

**Fig. 4:**
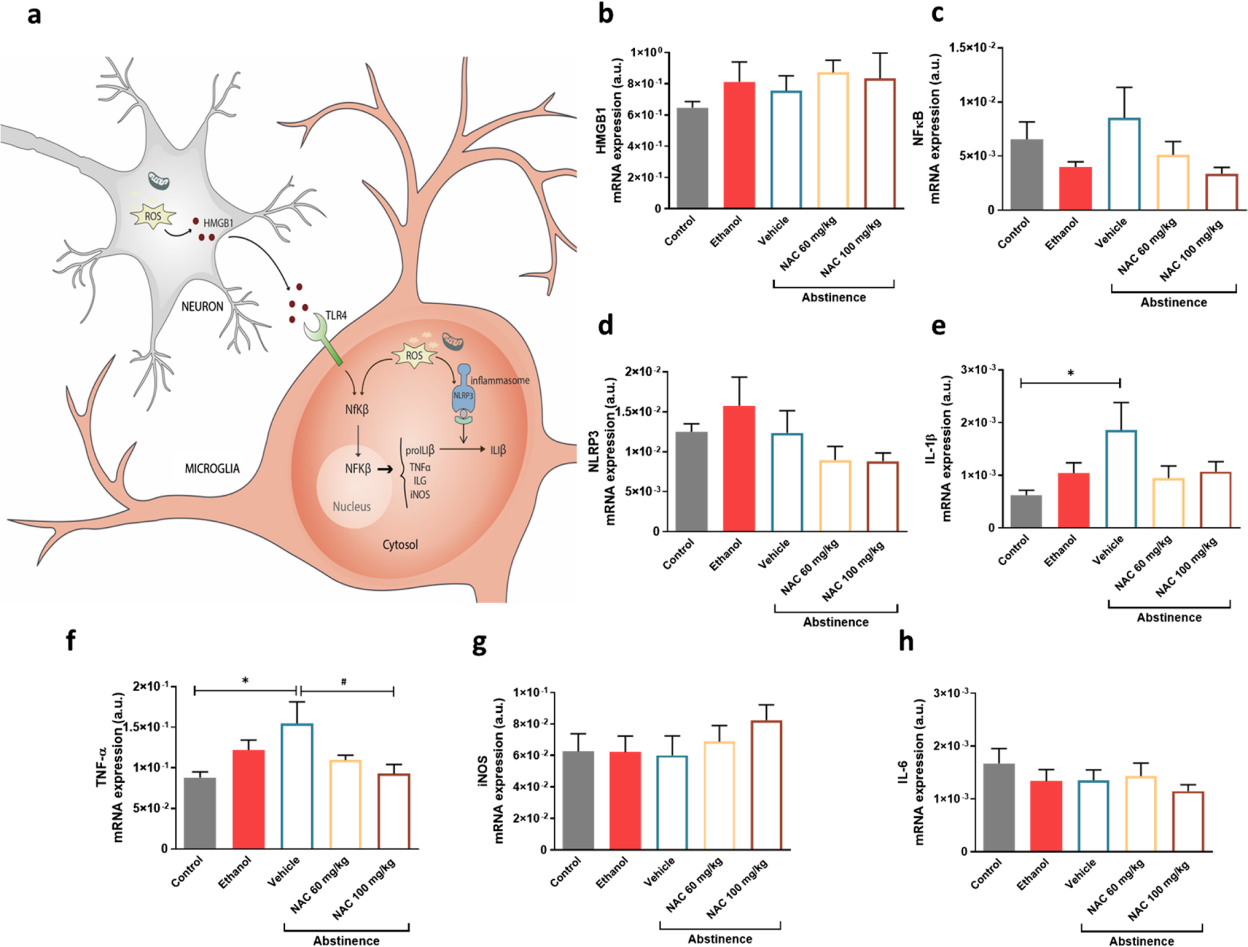
Neuroinflammatory mediators in prefrontal cortex after (i) chronic ethanol intake, (ii) prolonged abstinence and (iii) effect of NAC treatment during prolonged abstinence. (**a**) Schematic representation of the potential neuroinflammatory mediators interaction in neuron (grey) and glia (orange). mRNA levels (expressed in arbitrary units) of HMGB1 (**b**), NfKβ (**c**), NLRP3 (**d**), IL-1β (**e**), TNFα (**f**), iNOS (**g**) and IL-6 (**h**). Each graph represents mRNA levels from rats under the different experimental conditions described in Fig. 1. The colour legend is the same as the one detailed in Fig. 2. Data are represented as mean ± SE. For data analysis, two different one-way ANOVA followed by Tukey’s post hoc were applied. Asterisk (*p < 0.05) indicates significant differences among “control”, “ethanol” or “vehicle” groups and the hash symbol (^#^*p* < 0.05) indicates significant differences among “vehicle”, “NAC 60 mg/kg” or “NAC 100 mg/kg groups

The neuroinflammatory status in PFC after chronic ethanol exposure and during a protracted abstinence in the absence or presence of NAC treatment was also explored using our preclinical model. For this purpose, mRNA levels of various inflammatory modulators/mediators, including HMGB1, NfKβ, NLRP3, IL-1Β, TNFα, iNOS and IL-6, in the PFC of all animals were analysed. Results derived from the control and ethanol group were previously reported and discussed (Fernández-Rodríguez et al. 2022). These data have been plotted and included in the statistical analysis of the present paper for comparative purposes (Fig. 4). Statistical analysis only revealed differences in RNA levels in the case of IL-1Β (F2,14) = 4.322; *p* = 0.0345) and TNFα (F(2,13) = 4.517; *p* = 0.0320). As can be observed in Fig. 4e, rats under abstinence presented a significant increase in the IL-1Β value (around 150%) with respect to the control group (*p* = 0.0287). Treatment with either 60 or 100 mg/kg NAC during that period clearly tends to restore IL-1Β levels, although statistical analysis was not able to detect significant differences when compared to the vehicle group (*p* = 0.1944 and *p* = 0.2809, respectively) (Fig. 4e). A similar trend was observed in the case of TNFα, i.e., levels of this mediator increased during the abstinence period when compared with the control group (*p* = 0.0260). However, in this case, treatment with 100 mg/kg NAC was able to abolish the increase (*p* = 0.0270, with respect to the vehicle group) (Fig. 4f). mRNA levels of HMGB1, NfκB, NLRP3, iNOS and IL6 remained unaltered in PFC under our experimental conditions (Fig. 4b, c, d, g and h). Finally, it is worth to mention that the NfKβ levels tended to rise during the abstinence period and, again, NAC seems to blunt these values. Nevertheless, statistical analysis did not detect significant differences.

## Discussion

The present study provides evidence that neurobiological alterations related to brain oxidative stress and neuroinflammation observed after 3 weeks of ethanol abstinence in long-term ethanol-experienced male rats, can be alleviated by chronic NAC administration during abstinence. Conversely, NAC does not seem to blunt the modifications observed in some astrocytic glutamate transporters (downregulation of GLT-1 and GLAST in Nacc) during abstinence under the same experimental conditions. One of the most outstanding aspects of the present study is that our findings show evidence of the mechanism underlying the anti-relapse effects of NAC during abstinence in an animal model of high face and predictive validity (Bell et al. 2017; Leong et al. 2018).

### Effect of chronic ethanol intake and abstinence on the glutamate transporters expression

Our results provide evidence of the ethanol actions in the glutamate transporters expression and confirm other studies showing alterations in the GLT-1- and xCT expression in different models of chronic ethanol consumption (Sari et al. 2013; Alhaddad et al. 2014; Das et al. 2015; Hakami et al. 2016; Ezquer et al. 2019). The potential alteration of GLAST levels under chronic ethanol intake has been studied to a lesser extent (Alhaddad et al. 2014; Ezquer et al. 2019; Hakami et al. 2016). Furthermore, the results found in the literature on the effects of alcohol on GLT-1- and xCT expression are not fully coincident. The high variability and discrepancies reported are probably related to different variables such as rat strains, the use of ethanol preferent vs naïve animals and, particularly, the ethanol administration paradigm used (Abulseoud et al. 2014). In relation to long term ethanol exposition protocols, intermittent access to alcohol (IAA) and relapse-like ethanol drinking seem to lead to different adaptations in GLT-1 and xCT in Nacc when compared to continuous access to ethanol. Thus, in male P rats with continuous access to ethanol in their home cage, a downregulation of GLT-1 expression has been consistently reported (Sari et al. 2013; Alhaddad et al. 2014; Hakami et al. 2016)). However, when an IAA paradigm or the paradigm that alternates abstinence with ethanol intake phases was used, no changes in GLT-1 expression were observed in rats and mice (Pati et al. 2016; Stennett et al. 2017; Hammad et al. 2021; Das et al. 2022; Griffin et al. 2021; Ezquer et al. 2022). Similar results were observed in the case of xCT levels (Ding et al. 2013; Hakami et al. 2016). Regarding GLAST expression, no changes were observed in male P rats that have continuous access to ethanol (Alhaddad et al. 2014; Hakami et al. 2016) while reduced expression levels were reported when female P rats were used (Ding et al. 2013). Overall, an in-depth analysis of the above data shows that chronic ethanol intake provokes a decrease or no alteration of expression levels of these Glu transporters. This lack of effect is in accordance with the present findings showing no modification in GLT-1, GLAST and xCT levels in Nacc of naïve male rats after chronic alcohol exposure. According to the literature focused on glutamate homeostasis during the withdrawal period, the results are scarce and controversial. For instance, early abstinence conditions were reported not to affect GLT-1 and GLAST expression in Nacc (Melendez et al. 2005; Pati et al. 2016), but to downregulate xCT expression (Peana et al. 2014; Lebourgeois et al. 2019). The only study found analysing the consequences of a 2-week abstinence period, showed no alteration in GLT-1 and xCT protein expression, while GLAST levels were found to be diminished in Nacc of female P rats (Ding et al. 2013). Here, we provide the first evidence of how long-term voluntary ethanol drinking with repeated deprivation periods affects the expression of GLT-1, GLAST and xCT proteins during long-term abstinence. Concretely, a significant reduction in GLT-1 levels in Nacc during a protracted abstinence was found (around 45%) with respect to the control and ethanol groups. In the case of GLAST expression, a decrease was also detected as a consequence of a protracted abstinence, showing a similar trend to that observed in GLT-1. However, no changes in xCT levels were detected under any experimental condition. In summary, we observed a decrease in some of the glutamate uptake protein levels in Nacc that concur with the largest concentration of synaptic glutamate levels reported during prolonged abstinence in this brain area (Griffin et al. 2015; Pati et al. 2016).

Concerning DS, we selected this area because it is not only involved in the progressive development of compulsive drug seeking but also in the loss of control over drug intake (Belin and Everitt 2008; Vollstädt-Klein et al. 2010). Moreover, Ducret et al. reported that cocaine intake induced a decrease in GLT-1 expression in this area and NAC was able to reverse it (Ducret et al. 2016). In the case of ethanol, glutamate homeostasis has been explored to a lesser extent in DS. According to our results, neither chronic ethanol consumption nor prolonged abstinence caused any changes in the expression levels of any of the assayed glutamate transporters in comparison to the control group. These results are partially in accordance with the scarce data found in the literature. Thus, Morais-Silva et al. demonstrated no changes in xCT expression in the caudate area of mice which had received 2 g/kg/day ethanol i.p. injections for 13 days (Morais-Silva et al. 2016). Regarding the abstinence effect, Abulseoud et al. showed a decrease in GLT-1 in total striatum after 7 days of withdrawal (Abulseoud et al. 2014). Our results also showed that NAC administration was not able to significantly alter the levels of any of the tested transporters.

Taken together, the present findings provide new experimental data in the field of altered glutamate homeostasis and SUD. Moreover, our data, obtained under a preclinical model of voluntary ethanol intake and repeated abstinence, offers a more complete view of how repeated ethanol abstinence could be affecting glutamate transmission in AUD patients.

### Anti-relapse effect of NAC: evaluating the underlying mechanisms of action

The underlying mechanisms involved in the various phases of AUD development are not only multiple but also complex. Although a wealth of studies has highlighted the role of glutamate homeostasis in relapse (Kalivas 2009) in the last decades, research has started to identify alterations in neuroimmune signalling associated with addiction-related behaviours, including relapse (Knapp et al. 2016; Berríos-Cárcamo et al. 2020; Gipson et al. 2021; Namba et al. 2021). On the other hand, the duration of alcohol exposure and withdrawal are important factors when considering alcohol-induced changes in the neuroimmune function. Oxidative stress and neuroinflammation are tightly connected and the existence of a drug-induced brain oxidative stress-neuroinflammation vicious cycle, which can start from either one of its components or often by both, has been proposed (Berríos-Cárcamo et al. 2020). Additionally, mounting evidence suggests interactions of neuroimmune mechanisms with known drug-induced changes in the glutamate system (see review Gipson et al. 2021). These pivotal observations have led to the suggestion of several biological molecules and chemical agents to be used as improved treatments to reduce drug consumption as well as prevent relapse (Berríos-Cárcamo et al. 2020; Gipson et al. 2021; Namba et al. 2021). Among these compounds, NAC is one of the most studied.

Although there are several studies investigating the potential mechanism of action of NAC in the prevention of ethanol-motivational properties, ethanol-seeking or ethanol binge-like behaviour (Schneider et al. 2015; Morais-Silva et al. 2016; Quintanilla et al. 2018; Lebourgeois et al. 2019; Israel et al. 2021), there is a lack of studies analysing its effect during abstinence (i.e., when ethanol cannot interfere). To address this gap, our study is focused on the NAC effects on glutamate transporter expression, oxidative status parameters and neuroinflammation markers that potentially could be altered by ethanol withdrawal.

#### Effect of NAC treatment during abstinence on glutamate transporter expression

NAC capability to upregulate GLT-1 and xCT levels altered by cocaine consumption has been repeatedly demonstrated and proposed as a potential mechanism of action (Knackstedt et al. 2010; Reissner et al. 2015; Ducret et al. 2016). Nonetheless, the ability of NAC to alter protein expression after chronic ethanol intake is less clear. The present results have shown that NAC did not have any effect on GLT-1, GLAST nor xCT expression, i.e., in our rats, with a long-term drinking history with several deprivation period, NAC treatment during abstinence was not able to reverse glutamatergic alterations observed in Nacc. Consequently, our reported anti-relapse effect of NAC (Cano-Cebrián et al. 2021) does not seem to be linked with its effect on Glu transporters expression. Nonetheless, it is important to highlight that our previous data demonstrated an impairment of oxidative stress status during abstinence that, according to the present results, is alleviated by NAC treatment. On the other hand, the literature indicates that GLT-1, GLAST and system xCT activities are altered by oxidant environment (Trotti et al. 1988; Lewerenz et al. 2006). Therefore, there is the possibility that the anti-relapse effect of NAC be mediated through the alteration of the activity of these proteins rather than of the amount of the transporter (Stennett et al. 2017; Ezquer et al. 2022). Further research is needed to elucidate if the activity or function of GLT-1, GLAST or xCT is affected in our experimental conditions during ethanol abstinence and if NAC could restore it.

#### Effects of NAC on oxidative status altered by ethanol withdrawal

The studies analysing the NAC involvement in protection against oxidative damage in brain during abstinence are scarce. The current study has tried to address, at least in part, this issue by determining the potential beneficial effects of chronic NAC treatment during abstinence in oxidative stress caused by protracted ethanol withdrawal. It is worth mentioning that our recently reported results clearly suggest an impairment in oxidative balance during abstinence in Hip but not in AMG (Fig. 3) (Fernández-Rodríguez et al. 2022). NAC treatment during abstinence at both doses (60 and 100 mg/kg) was able to restore altered oxidative stress indicators. Similarly, Mocellin and collaborators demonstrated that treatment with NAC was able to protect the brain of zebrafish from the oxidative damage provoked by ethanol withdrawal after repeated exposure (Mocelin et al. 2019). In the same line, Akhtar et al. reported that ethanol-induced protein oxidation at 24 h following an episode of ethanol exposure in pregnant mice was completely prevented by supplementation with NAC in both fetal and maternal brain (Akhtar et al. 2017). Remarkably, our experimental conditions are more distant from those used in zebrafish or in pregnant mice, but, closer to the clinical setting, i.e., is closer to what occurs with alcoholic patients. In fact, the animal model used tries to encompass the entire range of the addiction cycle, including acquisition and maintenance of drug taking, withdrawal and craving during periods of drug abstinence and ultimately relapse; processes that were repeated several times (Leong et al. 2018). Nonetheless, generally, the results obtained in the different studies point in the same direction and underline the importance of analysing alterations in oxidative status during abstinence, i.e., without the interference of ethanol and the role of NAC in preventing them.

### Effects of NAC on neuroinflammation markers altered by ethanol withdrawal

Oxidative stress and inflammation are tightly related in a positive loop (Berríos-Cárcamo et al. 2020). Under our experimental conditions, NAC treatment was able to normalize the altered oxidative status determined in rat Hip. In the PFC, our results also showed an increase in IL1β and TNFα expression during abstinence that is prevented by NAC in the case of TNFα. In relation to NfκB, an appreciable increase during abstinence can also be observed that seems to be alleviated by NAC, although statistical analysis was not able to detect significant differences. All these observations suggest that reactive oxygen species (ROS) could directly enhance NfKβ activity and promote IL1β and TNFα expression in PFC during abstinence (Fig. 4), but NAC presence prevents the impairment in ROS levels and the subsequent increase in proinflammatory markers. To test this possibility, it would be very interesting to elucidate if ROS levels are also increased in the PFC during abstinence, but the limited amount of biological material available impeded us from performing both determinations in the same sample. Further experiments are warranted to delve into this point.

Overall, our data provide new information about neuroinflammatory pathways in PFC of non-preferent rats that have experienced several abstinence periods and the NAC anti-inflammatory effect under these conditions. Remarkably, few studies have attempted to manipulate neuroimmune mechanisms experimentally using drug self-administration animal models. Hence, our study has made the first steps to address a remaining gap in the field in agreement with the proposals of other authors (Namba et al. 2021).

#### NAC treatment during abstinence seems to mimic the effects displayed by ethanol re-introduction on oxidative and neuroinflammatory status

An interesting aspect reported in our previous research was the role of ethanol re-introduction at several neurobiological levels such as oxidative and neuroinflammation status (Fernández-Rodríguez et al. 2022). Indeed, when ethanol was reintroduced after a protracted deprivation period, it was able to restore increased GSSG/GSH levels as well as neuroinflammatory markers such as IL-1ß and TNF-α. Remarkably, according to the present results, NAC seems to mimic some of these ethanol effects. An in-depth analysis of both effects, i.e. ethanol re-introduction and NAC administration during abstinence, show a similar profile. Hence, as can be seen in Table 2, in the case of oxidative status, NAC doses provoked a reduction of around 65% in GSSG/GSH levels in relation to the abstinence (vehicle) group, whereas ethanol reintroduction induced an 89% decrease. Regarding inflammatory mediators, the results were qualitatively similar, with the percentage of reduction being less marked for both NAC and ethanol, than in the case of oxidative stress markers. In all cases, ethanol effect is always more pronounced than that of NAC. Consequently, it could be hypothesized that during abstinence, NAC administration could exert the same role than ethanol reintroduction, thus reducing the propensity for ethanol consumption relapse. Additionally, our group have also demonstrated that NAC, acutely administered, was capable to mimic some of the actions in the mesocorticolimbic system provoked by the acute administration of ethanol (Fernández-Rodríguez et al. 2021). These striking data suggest that further experiments using more mechanistic approaches are needed to decipher the role of NAC to prevent neurobiological alterations induced during relapse.

**Table 2:** Comparative effects of NAC (60 or 100 mg/kg) and ethanol re-introduction on oxidative stress and neuroinflammatory markers

		Percentage of reduction in comparison with vehicle (abstinence) group		
	Parameter	NAC 60 mg/kg	NAC 100 mg/kg	Ethanol re-introduction
Oxidative stress	GSSG	61%*	62%*	89%*
	GSSG/GSH	65%*	66%*	84%*
Inflammatory markers	IL-1β	53%	42%	79%*
	TNF-α	30%	40%*	56%*

(*) Denotes statistical differences with respect to the abstinence group. In the case of NAC treatment, the statistical results were obtained from Figs. 3 and 4. In the case of ethanol re-introduction, statistical results come from our previous report (Fernández-Rodríguez et al. 2022) (* *p* < 0.05).

## Conclusions

This study provides evidence, by using an animal model of high face and predictive validity, that the NAC mechanism of action that underlies its anti-relapse effect (Cano-Cebrián et al. 2021) is probably related to both its antioxidant and anti-inflammatory capabilities. Although preclinical findings suggest that the NAC may assist the treatment of SUD through direct effects on cysteine/glutamate exchange (Gipson 2016; Kalivas and Kalivas 2022), the present data indicate that NAC anti-relapse effect, in our preclinical model, does not seem to be related to alterations of Glu protein expression levels. Further research is needed to deeply elucidate NAC action in abstinence. Testing the activity of glutamate transporters and system xCT and the analysis of oxidative stress and neuroinflammation in the same areas will also provide relevant information. These are relevant future research areas which would allow the optimisation of existing pharmacotherapies and the identification of new targets.

## Data Availability

Raw data from published studies is available to investigator upon request.
